# High-resolution electrical and chemical characterization of nm-scale organic and inorganic devices

**DOI:** 10.3762/bjnano.4.35

**Published:** 2013-05-16

**Authors:** Pierre Eyben

**Affiliations:** 1 IMEC VZW, Kapeldreef 75, 3001 Leuven, Belgium

Almost ever since the advent of the microelectronics adventure, silicon-based MOSFET (metal–oxide–semiconductor field-effect transistor) technology has been largely dominant. However, for a few years now this technology has exhibited some fundamental limitations in tackling the increasingly challenging issues of miniaturization and improvements in processing speed and power consumption. Hence, new inorganic semiconductor materials (Ge, InGaAs, InP, InSb, GaN, GaSb, SiC, etc.) and new architectures (multiple-gate FETs, nanowire T-FETs, etc.) are being progressively introduced.

These new materials are mainly introduced due to their increased mobility (to boost the processing speed of the devices), and the new architectures are required to reduce the junction leakage (and hence reduce the power consumption). These architectures are typically three-dimensional, and with the continuous decrease of dimensions, they also represent extremely confined volumes in which statistical and quantum effects start to play an increasing role. The entirely successful application of these new materials and architectures towards realizable technologies is facing some challenges. The most significant one is probably the impact of defects linked to growth processes and to the presence of stress. Indeed, the presence of different semiconductor materials with different lattice dimensions leads to crystalline defects, threading dislocations, and microtwins that affect the diffusion of dopants and the material mobility (due to scattering). When growth is performed in narrow trenches, dislocations are trapped within the confined volume (aspect-ratio trapping) and, theoretically, defect-free layers can be obtained. However, even if an apparently defect-free layer is obtained, the polar nature of the complex compound materials implies that antiphase boundaries can still be formed, which potentially represent important charge and recombination centers.

Beyond the standard logic/memory applications there is a very strong increase in “More than Moore” developments targeting energy (photovoltaic, energy storage), imaging (e.g., quantitative medical imaging), sensor/actuators linked to CMOS-base circuitry, biochips, etc. The utilization of graphene in order to process high mobility (both for holes and electrons) field-effect transistors is also being intensively studied.

In all these cases, metrology is a challenge, and no universal solution is identifiable. Moreover in many cases it becomes also very difficult to establish a complete assessment of accuracy, precision and spatial resolution due to a lack of appropriate 3-D standards and comparative metrology.

The development of high-performance devices (high speed and density, low consumption) is not the only objective of the electronic industry. The need for low-cost devices processed industrially on flexible and light substrates over very large surfaces has led to the emergence of electronic components based on organic semiconductors. The organic materials used nowadays are typically made of single molecules in highly ordered assemblies or of polymeric semiconductors in thin films. In recent years, an extensive set of organic-based prototypes (transistors, sensors, electrochromic devices, biosensors, photodiodes, photovoltaic cells, etc.) have been developed, demonstrating the strong potential of these materials. However, the advent of commercial applications often requires important breakthroughs towards more efficient and stable organic photovoltaic devices. This implies reduced exciton diffusion lengths (and thus more efficient collection) through the fabrication of an entangled mixture of the acceptor–donor layers, the addition of light scattering nanoparticles or metallic nanoparticles (spectrum harvesting through plasmonics) in the active layer or even wavelength convertors based on metal nanoparticles with a dye to shift the wavelengths. Improving the lifetime of organic solar cells requires incorporating optically transparent inorganic barriers in between the polymer films to prevent moisture penetration.

Unfortunately, these organic systems represent an even more challenging metrological problem as compared to their inorganic counterparts, as it no longer suffices to determine the atomic and electrical distributions, but one is also faced with the additional, equally important problem to trace, on the nanometer scale, the chemical configuration and the polymeric information.

It is therefore important to develop and improve two- and three-dimensional characterization techniques that can be utilized on both organic and inorganic semiconductors. These techniques should allow determination of the carrier/dopant distribution with an excellent sensitivity and repeatability (within 3 to 5%) with nanometer spatial resolution (subnanometer in inorganic and below 10 nm in organic) over a broad dynamic range (up to five decades). Ideally, they should also be able to probe the elemental distribution and to provide information on chemical bonding.

In this Thematic Series, we present the work of various leading labs in developing such techniques. Targeting 2-D/3-D resolution, one inevitably needs to look at scanning probe techniques that can be proclaimed to be dominant for electrical characterization and atomic probes that can be viewed as the ultimate in terms of compositional analysis.

At the same time we need to recognize that, notwithstanding the past efforts and achievements, there remains a considerable knowledge and performance gap between the state-of-the-art metrology and the future metrology requirements. Hence also some simulation work is presented in this Thematic Series.

Pierre Eyben

Leuven, April 2013

